# Single oral fixed-dose praziquantel-miltefosine nanocombination for effective control of experimental schistosomiasis mansoni

**DOI:** 10.1186/s13071-020-04346-1

**Published:** 2020-09-15

**Authors:** Maha M. Eissa, Mervat Z. El-Azzouni, Labiba K. El-Khordagui, Amany Abdel Bary, Riham M. El-Moslemany, Sara A. Abdel Salam

**Affiliations:** 1 Department of Medical Parasitology, Faculty of Medicine, Alexandria University, Alexandria, Egypt; 2 Department of Pharmaceutics, Faculty of Pharmacy, Alexandria University, Alexandria, Egypt; 3 Department of Pathology, Faculty of Medicine, Alexandria University, Alexandria, Egypt

**Keywords:** Praziquantel, Miltefosine, Lipid Nanocapsules, *Schistosoma mansoni*, Nanocombination, Multistage activity, Tegumental targeting, Scanning electron microscopy

## Abstract

**Background:**

The control of schistosomiasis has been centered to date on a single drug, praziquantel, with shortcomings including treatment failure, reinfection, and emergence of drug resistance. Drug repurposing, combination therapy or nanotechnology were explored to improve antischistosomal treatment. The aim of the present study was to utilize a novel combination of the three strategies to improve the therapeutic profile of praziquantel. This was based on a fixed-dose nanocombination of praziquantel and miltefosine, an antischistosomal repurposing candidate, co-loaded at reduced doses into lipid nanocapsules, for single dose oral therapy.

**Methods:**

Two nanocombinations were prepared to provide 250 mg praziquantel-20 mg miltefosine/kg (higher fixed-dose) or 125 mg praziquantel-10 mg miltefosine/kg (lower fixed-dose), respectively. Their antischistosomal efficacy in comparison with a non-treated control and their praziquantel or miltefosine singly loaded counterparts was assessed in murine schistosomiasis mansoni. A single oral dose of either formulation was administered on the initial day of infection, and on days 21 and 42 post-infection. Scanning electron microscopic, parasitological, and histopathological studies were used for assessment. Preclinical data were subjected to analysis of variance and Tukeyʼs *post-hoc* test for pairwise comparisons.

**Results:**

Lipid nanocapsules (~ 58 nm) showed high entrapment efficiency of both drugs (> 97%). Compared to singly loaded praziquantel-lipid nanocapsules, the higher nanocombination dose showed a significant increase in antischistosomal efficacy in terms of statistically significant decrease in mean worm burden, particularly against invasive and juvenile worms, and amelioration of hepatic granulomas (*P* ≤ 0.05). In addition, scanning electron microscopy examination showed extensive dorsal tegumental damage with noticeable deposition of nanostructures.

**Conclusions:**

The therapeutic profile of praziquantel could be improved by a novel multiple approach integrating drug repurposing, combination therapy and nanotechnology. Multistage activity and amelioration of liver pathology could be achieved by a new praziquantel-miltefosine fixed-dose nanocombination providing 250 mg praziquantel-20 mg miltefosine/kg. To the best of our knowledge, this is the first report of a fixed-dose nano-based combinatorial therapy for schistosomiasis mansoni. Further studies are needed to document the nanocombination safety and explore its prophylactic activity and potential to hinder the onset of resistance to the drug components.
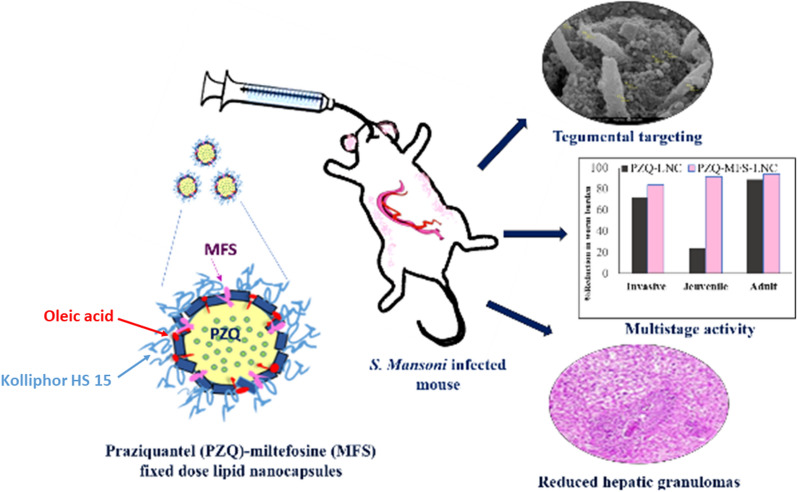

## Background

Schistosomiasis is a debilitating parasitic disease affecting more than 200 million people over 78 tropical and subtropical countries, of which a significant number are preschool children [1, 2]. The disease is caused by three main species of the genus *Schistosoma* having complex life-cycles that involve an intermediate snail host and a definitive human host. *Schistosoma mansoni*, the causative agent of intestinal schistosomiasis, is acquired by human contact with freshwater containing infectious cercariae. Egg deposition in the liver is associated with the formation of granulomas and subsequent serious complications [1]. In addition, both adult schistosomes and deposited eggs induce immunomodulatory effects that impair the host immune defences against other pathogens, such as human immunodeficiency and hepatitis B/hepatitis C viruses [3, 4].

No effective vaccine exists to date and chemopreventive therapy of schistosomiasis has relied for decades on praziquantel (PZQ), a low-cost well-tolerated drug with proven efficacy against the major species of schistosomiasis. According to the World Health Organization, PZQ is administered at the standard single oral dose of 40 mg/kg body weight in mass drug administration (MDA) programmes in endemic countries. Although such campaigns resulted in reduction of worm burden, incomplete cure attributed to ineffectiveness of PZQ against immature juvenile worms and reinfection have been reported [5, 6]. Moreover, drug resistance due to the widespread use of PZQ presents a serious threat to the gains achieved [7].

Taking these concerns into consideration and given the modern resources of drug development, synthesis of PZQ derivatives [8], discovery of alternative lead compounds [9, 10], natural products [11, 12] and repurposing of existing drugs [13, 14] are effective approaches for the introduction of new antischistosomal agents. Recently, we demonstrated that miltefosine (MFS), a membrane active alkylphosphocholine, is a promising repurposing candidate against schistosomiasis [13, 15]. The drug showed multistage activity in five successive 20 mg/kg/day oral doses in mice which could be significantly enhanced by lipid nanoencapsulation, allowing for a 20 mg/kg single dose oral therapy [16, 17].

Nevertheless, building on the clinical and pharmacoeconomic merits of PZQ, more success can be achieved by overcoming the main limitations of PZQ therapy. Application of new strategies, such as nanotechnology and combinational therapy may extend the useful life of PZQ in more effective new formulations. In this respect, pharmaceutical nanotechnology may address challenges such as inadequate solubility, bioavailability, cellular delivery as well as non-specific biodistribution and rapid clearance of antiparasitic drugs [18, 19]. Indeed, drug delivery systems including lipid-based nanocarriers such as liposomes [20] and solid lipid nanoparticles [21], niosomes [22] and silica nanoparticles [23] were shown to enhance the bioavailability and antischistosomal activity of PZQ. Recently, we demonstrated that entrapment of PZQ into lipid nanocapsules (LNCs) significantly enhanced its antischistosomal activity in a single oral reduced dose of 250 mg/kg in mice [24]. LNCs are nanostructures with great potentials in drug delivery [25–27]. Owing to their relatively small and controllable size (20–100 nm), structural integrity in simulated gastrointestinal (GI) fluids and possible active transport across the intestinal epithelium, LNCs are highly promising as oral nanovectors [28, 29]. In schistosomiasis treatment, oral LNCs also showed potential *S. mansoni* tegumental targeting [24].

Apart from nanotechnology, drug combination therapy aiming at synergies, resistance reduction and rejuvenation of old drugs, is another approach showing increasing benefits in the treatment of several diseases, particularly cancer [30, 31] and bacterial infections [32]. In experimental schistosomiasis, promising results have been reported for combinations involving PZQ and other drugs or biomolecules, aiming at multistage targeting, amelioration of infection-associated pathologies and resistance reduction [12, 14]. Despite evident advantages, combinations of free drugs may display variation in the pharmacokinetics and membrane transport among component drugs in addition to intricate dosing, resulting in inadequate outcomes [33]. Such limitations led to the emergence of an innovative combination therapy approach based on multi-drug delivery nanocarriers with increasing benefits in diverse diseases, notably cancer [34, 35]. Although still in an early stage in the treatment of infectious diseases [36], the carrier-mediated multiple drug approach proved promising in the treatment of malaria [37, 38].

To date, the research on nanocarrier-mediated drug delivery against schistosomiasis has focussed mainly on monotherapy [39]. Thus, the objective of the present study was to improve the therapeutic profile of PZQ by overcoming its shortcomings, mainly ineffectiveness against immature worms and inadequate liver protection. In addition, combination therapy could have the potential to overcome the problem of reinfection as well as offer protection of the component drugs from development of resistance. Therefore, PZQ was combined with MFS with proven activity against all developmental stages of the parasite and liver protective effects. To allow single dose oral administration, imperative in MDA campaigns, without the intricate dosing and varying outcomes of free drug combinations, PZQ and MFS were co-loaded in LNCs as a single oral fixed-dose LNC nanocombination. The antischistosomal efficacy of the nanocombination was assessed at two dosing levels of both drugs in comparison with singly loaded PZQ LNCs in experimental schistosomiasis mansoni in mice. Scanning electron microscopy (SEM), parasitological parameters, and histopathological examination were utilized for antischistosomal efficacy assessment against the invasive, juvenile, and adult developmental stages of the parasite.

## Methods

### Materials

The following chemicals were used in the study: PZQ (high purity, gift of the Egyptian International Pharmaceuticals Industries Company (EIPICO), Cairo, Egypt); MFS (98–102%, Chem-Impex International, New York, USA); Labrafac® lipophile WL 1349 (Gattefossé SA, Saint-Priest, France); Kolliphor HS 15 (BASF, Ludwigshafen, Germany); Lipoid S100 (a soybean lecithin containing 94% of phosphatidylcholine, Lipoïd GMBH, Ludwigshafen, Germany); Oleic acid (OA, > 93%, Sigma-Aldrich Co., St Louis, MO, USA); acetonitrile HPLC grade (Thermo Fisher Scientific, Waltham, MA, USA); and Span® 80 (extra pure, LobaChemie for Laboratory Reagents and Fine Chemicals, Mumbai, India). All other chemicals were of analytical grade.

### Formulation and characterization of lipid nanocapsules

LNCs were formulated with oleic acid and Span 80 and prepared by the phase inversion method [24, 27]. In brief, Kolliphor® HS 15, Labrafac lipophile WL 1349 and deionized water containing NaCl (0.88% w/w of the final dispersion) were weighed and mixed using a magnetic stirrer in the ratio 5:6:9. Span 80 (2% w/w) and oleic acid (6 % w/w) were added to the primary mixture which was subjected to three progressive heating and cooling cycles between 45–75 °C at 4 °C/min. An irreversible shock was induced by a two-fold dilution with cold deionized water (0–2 °C) added to the formed o/w emulsion at a temperature 1–3 °C from the beginning of the phase inversion zone. This was followed by slow magnetic stirring at room temperature for 5 min. For the preparation of drug loaded LNCs, PZQ was added to the primary mixture of ingredients at concentrations 25 or 12.5 mg/ml, whereas MFS was added just before quenching at a concentration of 2 or 1 mg/ml of the final dispersion. The procedure was used to prepare a higher fixed-dose combination containing 25 mg PZQ and 2 mg MFS/ml of dispersion and a lower fixed-dose combination containing 12.5 mg PZQ and 1 mg MFS/ml of dispersion and their corresponding singly loaded counterparts, PZQ 25 mg/ml, MFS 2 mg/ml, PZQ 12.5 mg/ml and MFS 1 mg/ml. The fixed-dose combination LNC dispersions were used in calculated volumes to provide a dose of 250 mg/kg PZQ-20 mg/kg MFS and 125 mg/kg PZQ-10 mg/kg MFS respectively in the antischistosomal study in mice.

LNC formulations were characterized for morphology, colloidal properties, and drug entrapment efficiency (EE%). The morphology of LNCs was examined by transmission electron microscopy (TEM) using JEOL, JEM-100 CX electron microscope, Tokyo, Japan. Before analysis, the LNC dispersion was treated with 2% w/v uranyl acetate solution as a negative stain and sprayed onto copper grids. Shots were taken at ×5000 at 80 kV. The average particle size, polydispersity index (PdI) and zeta potential (ZP) were measured by photon correlation spectroscopy (PCS) at a fixed angle 173° using a 4 mW He-Ne laser at 25 °C. The EE% was obtained by determining the concentration of free (unentrapped) PZQ and MFS in the ultrafiltrate after separation of LNCs using an ultrafiltration/centrifugation technique. The concentration of unentrapped PZQ in the ultrafiltrate was determined by HPLC-UV as previously reported [24]. MFS concentration was measured by a modified spectrophotometric assay originally reported for quaternary ammonium compounds and validated for MFS quantitation [16].

### Antischistosomal efficacy in mice

#### *Schistosoma mansoni* infection of animals

The life-cycle of *S. mansoni* was maintained in the Medical Parasitology Department, Faculty of Medicine, Alexandria University by serial passages in laboratory-bred *Biomphalaria alexandrina* snails and Swiss albino mice [40]. A total of 128 mice, 6–8 weeks-old, weighing 20–30 g each, were obtained from the animal house of the Medical Parasitology Department, Faculty of Medicine, Alexandria University. Mice were housed under specific pathogen-free barrier conditions. Each mouse was infected with 100 ± 10 freshly shed cercariae using the paddling technique [41]. All mice were subjected to infection.

### Animal groups

A total of 128 mice were allocated to a non-treated control group (NT) including 8 mice and 2 experimental groups, Group I and Group II. Group I (*n* = 60) was subdivided into 3 subgroups as follows: Subgroup Ia, PZQ 250 mg/kg (*n* = 20); Subgroup Ib, MFS 20 mg/kg (*n* = 20); and Subgroup Iab, PZQ 250 mg-MFS 20 mg/kg (*n* = 20). Group II (*n* = 60) was subdivided into 3 subgroups as follows: Subgroup IIa, PZQ 125 mg/kg (*n* = 20); Subgroup IIb, MFS 10 mg/kg (*n* = 20); and Subgroup IIab, PZQ 125-MFS 10 mg/kg (*n* = 20).

Mice in all treated subgroups (Ia, Ib and Iab, IIa, IIb and IIab) were administered a calculated volume of the LNCs dispersion corresponding to the required dose of the PZQ-MFS fixed-dose combinations or the corresponding singly loaded LNCs by gastric gavage. Twenty mice in these subgroups were further subdivided into 3 subgroups (1, 2 and 3) which were given a single oral dose of the drug(s) nanoformulations at 3 different dates (on the initial day of infection, and days 21 and 42 post-infection (dpi)) corresponding to the three stages of *S. mansoni* life-cycle (invasive, juvenile and adult stages), respectively [17]. The number of mice/group treated on the initial day of infection and 21 dpi (against invasive and immature stages) was 6 whereas, it was 8 for all other subgroups treated on day 42 post-infection (p.i.) (against the adult stage). Two infected mice treated on day 42 p.i. were sacrificed 24 h after administration of the nanoformulations to collect the adult worms for morphological examination using SEM (Jeol JSM- IT200, Jeol, Tokyo, Japan). The remaining mice of all subgroups were sacrificed on day 49 p.i. The therapeutic efficacy of the two fixed-dose nanocombinations in comparison with the corresponding singly loaded control LNCs and the non-treated control was assessed by determination of the percentage reduction in total worm burden, the size of hepatic granulomas, histopathological changes in liver parenchyma and examination of the morphology of recovered worms by SEM.

### Antischistosomal activity assessment

#### Estimation of adult worm burden

On day 49 p.i., adult worms were recovered from the hepatic and mesenteric vessels from mice in all study groups using the perfusion technique [41].

#### Morphological examination by scanning electron microscopy

Adult worms were recovered 24 h post-administration of nanoformulations from two infected mice from all subgroups treated on day 42 p.i. against the adult stage for scanning electron microscopic (SEM) imaging. Worms were fixed in cold 2.5% buffered glutaraldehyde phosphate (pH 7.4), washed, dehydrated through an ascending series of ethanol, and left to dry in air. After drying, they were embedded in epoxy resin, mounted on aluminium stubs and coated with 20 nm gold particles in an ion-sputtering apparatus. Adult male worms were examined under SEM and photographed [42].

#### Histopathological examination

Samples of the liver of mice of all study groups were fixed in 10% neutral buffered formalin. Parrafin-embedded histological sections (5 μm-thick) were stained with hematoxylin and eosin (H&E) [43]. Pathological changes in the hepatic parenchyma were observed and the mean size of granulomas was determined. Only granulomas containing one central clearly identifiable egg were selected. Their diameters were measured with a light microscope, equipped with an ocular micrometer under low power magnification (100×). In each mouse, the mean diameter was calculated from 10 granulomata. Then, the mean granulomata size was estimated for each [24].

### Statistical analysis

Data were analysed using IBM SPSS software package version 20.0 (IBM Corp, Armonk, NY, USA). Kolmogorov-Smirnov test was used to verify the normality of distribution. Quantitative data were described using mean and standard deviation. Significance of the obtained results was judged at the 5% level. For normally distributed quantitative variables, ANOVA F-test was used to compare between more than two groups and Tukeyʼs *post-hoc* test was used for pairwise comparisons. The percentage reduction (% R) of the adult worm load as well as the granuloma size were calculated as follows:

$${\text{Percentage reduction }}\left( {{\text{\% R}}} \right) = \frac{{{\text{N}} - n }}{\text{N}} \times 100$$

where N is the mean number of worms or the mean granuloma size in the infected non-treated group and *n* is the mean number of worms or the mean granuloma size in the infected treated subgroups.

## Results

### Characterization of lipid nanocapsules

**Fig. 1:**
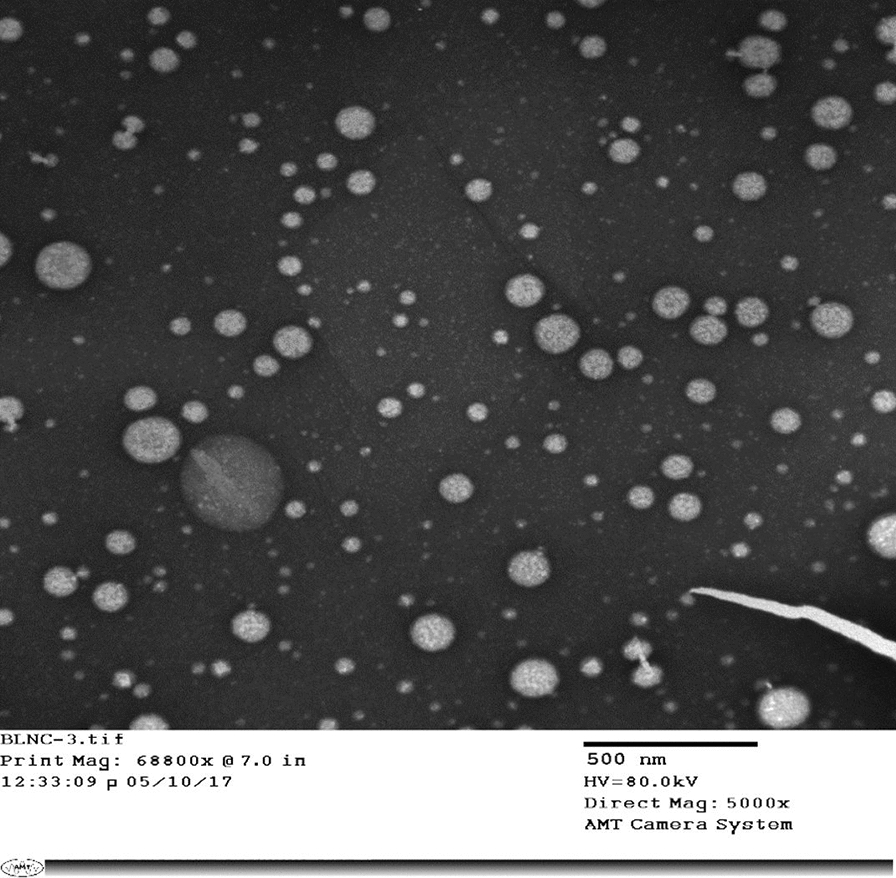
TEM image of praziquantel-miltefosine combination lipid nanocapsules

PZQ-MFS combination LNCs were prepared and characterized for size, PdI, ZP and EE% as reported earlier for PZQ LNCs [24]. TEM imaging of LNCs (Fig. 1) showed almost spherical nanostructures, homogenously distributed, and not aggregated. Characterization data verified small size and monodispersity (PdI not exceeding 0.05). Blank LNCs had a mean diameter of 52.2 ± 0.42 nm with a ZP of − 6.5 ± 0.4 mV. Drug loading into LNCs slightly but significantly affected their size and ZP (57.6 ± 0.15 nm and − 7.9 ± 0.4 mV, respectively) as indicated by the t-test (*t*_(4)_ = 20.972, *P* < 0.0001; *t*_(4)_ = 4.287, *P* = 0.0128, respectively). The EE% calculated based on drug content exceeded 97% for both PZQ and MFS.

### Antischistosomal activity in *S. mansoni*-infected mice

#### Assessment of adult worm burden

**Fig. 2:**
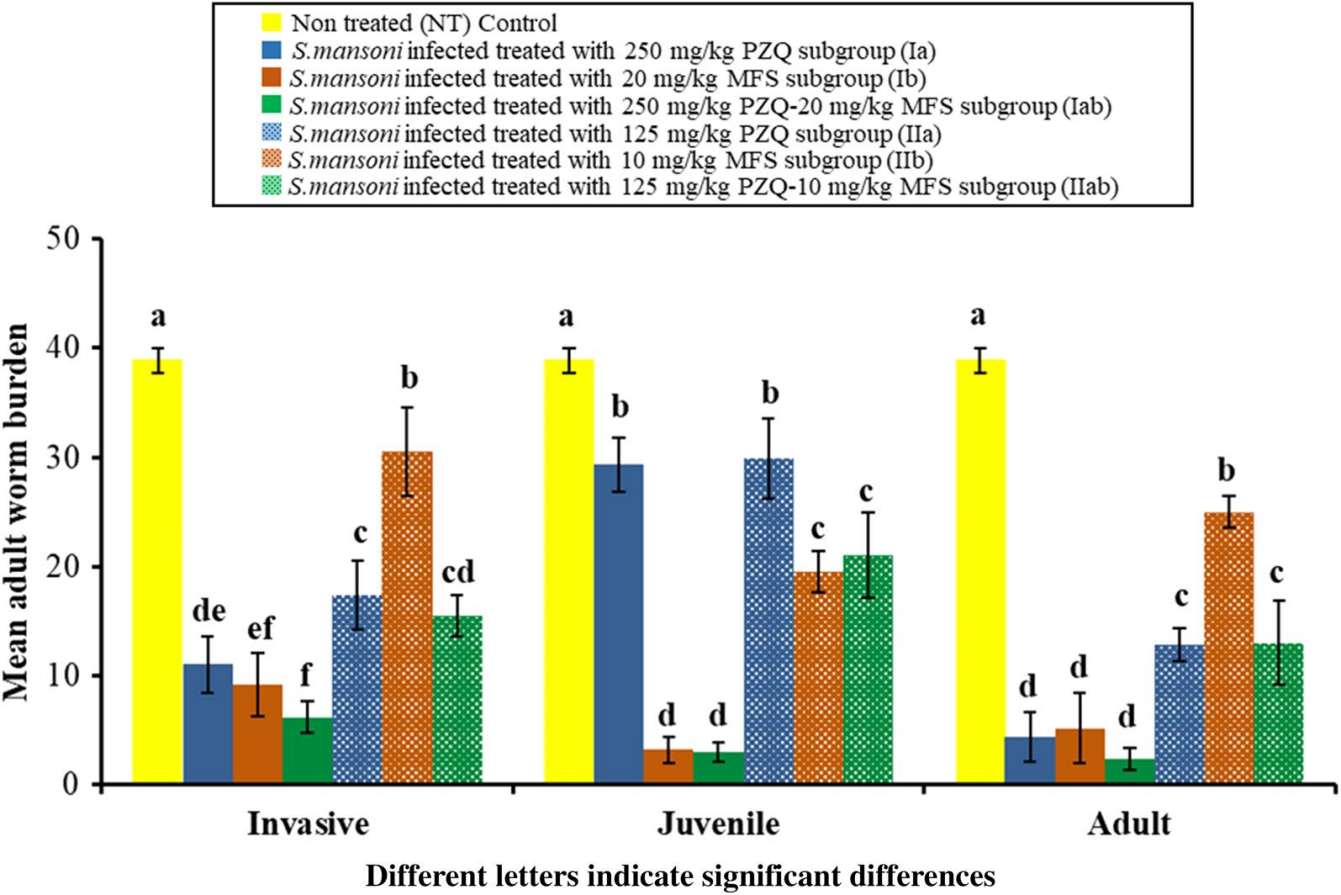
Effect of PZQ-MFS nanocombinations on worm burden in comparison with non-treated and respective monotherapy controls against different developmental stages

Data for worm burden assessment in the study subgroups are shown in Fig. 2. There was a statistically significant difference between the different studied subgroups against invasive (*F*_(6, 35)_ = 124.405, *P* < 0.001), juvenile (*F*_(6, 35)_ = 185.422, *P* < 0.001) and adult stages (*F*_(6, 35)_ = 197.693, *P* < 0.001). Oral administration of the two fixed-dose nanocombinations, 250 mg PZQ-20 mg MFS/kg (subgroups Iab 1, 2 and 3) and 125 mg PZQ-10 mg MFS/kg (subgroups IIab 1, 2 and 3) to infected mice given against invasive, juvenile and adult worms resulted in a statistically significant reduction in the mean adult worm burden of (6.17 ± 1.47, 3.0 ± 0.89 and 2.33 ± 1.03) and (15.50 ± 1.87, 21.0 ± 3.90 and 13.0 ± 3.90) for the two subgroups, respectively, compared to a mean of 38.83 ± 1.17 for the infected non-treated control subgroup (*P* < 0.001). On comparing the two different doses of PZQ-MFS LNCs (subgroup Iab and subgroup IIab) together, a statistically significant reduction in the mean worm burden was observed in favour of the higher nanocombination dose given against the three different developmental stages (subgroups Iab 1, 2 and 3) (*P* < 0.001). There was also a statistically significant reduction in the mean adult worm burden of mice treated with a higher dose of PZQ-MFS LNCs (subgroup Iab) compared with PZQ-LNCs monotherapy given against invasive and juvenile stages (subgroups Ia 1 and 2) (11.0 ± 2.61 and 29.33 ± 2.50, respectively) (*P* = 0.044; *P* < 0.001, respectively).

Statistical analysis of worm burden data of the two fixed-doses PZQ-MFS LNCs treated subgroups against the different developmental stages of *S. mansoni* worms compared to the infected non-treated control, corresponding monotherapy controls as well as to each other are shown in Fig. 2.

#### Morphological properties by SEM

**Fig. 3:**
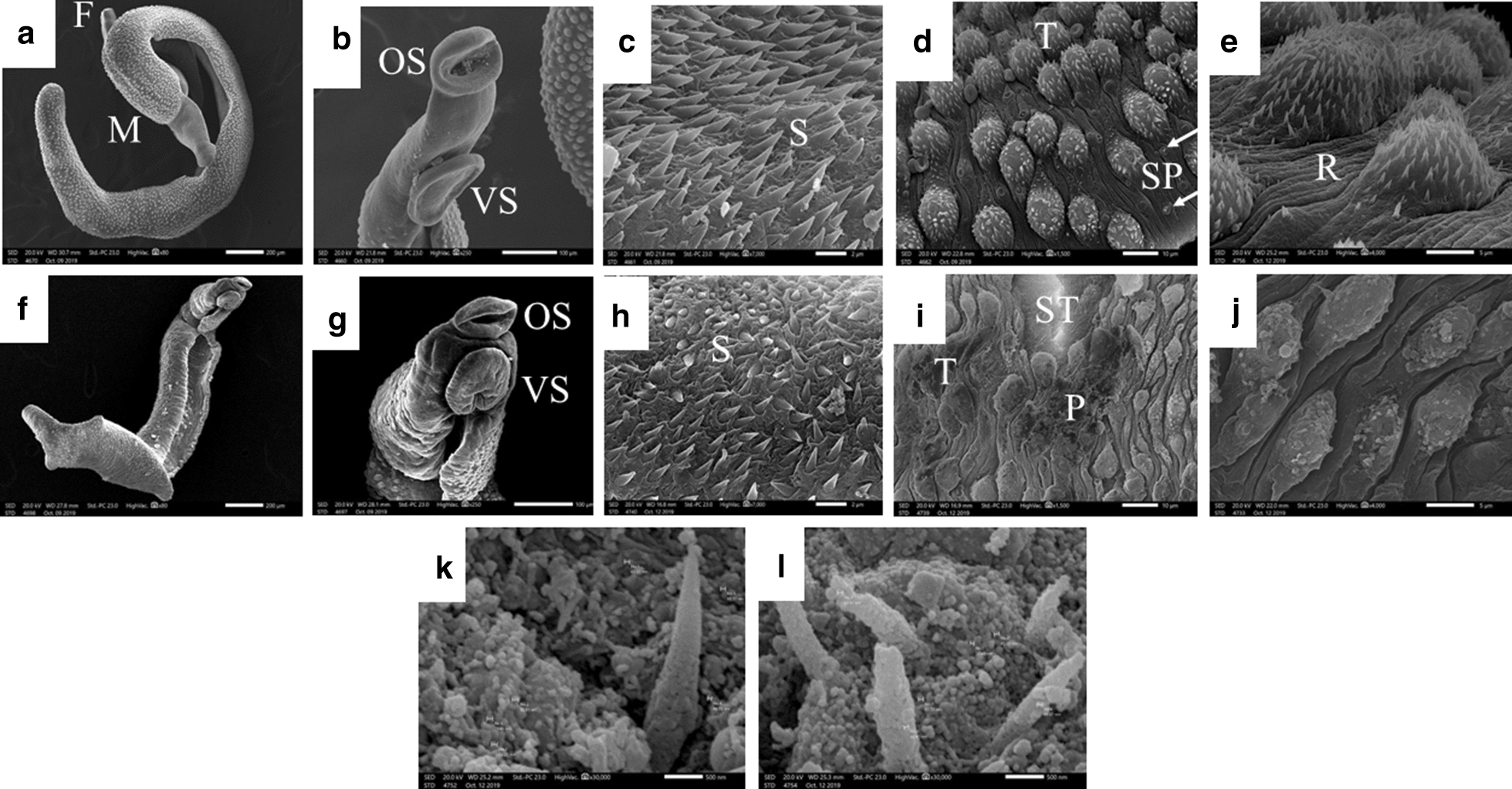
Scanning electron micrographs of *S. mansoni* male worms recovered from the hepatic and mesenteric veins of infected non-treated mice (**a**–**e**) and infected mice treated with the higher PZQ-MFS nanocombination dose (PZQ 250 mg-MFS 20 mg/kg, subgroup Iab) (**f**–**l**). **a** Normal male worm in copula with female (×80). **b** Normal oral and ventral suckers (×250) with apically directed spines (×7000) (**c**). **d** Normal dorsolateral tegument surface of the mid body showing crablike uniformly distributed tubercles with sharp visible intact spines and sensory papillae (×1500). **e** Normal tegumental ridges between the tubercles (×4000). Adult males recovered from *S. mansoni*-infected mice treated with the higher fixed-dose nanocombination (subgroup Iab) (**f**–**l**). **f** Deformed whole body (×80). **g** Deformed oral and ventral suckers (×250). **h** Blunt, short and loose spines (×7000). **i** Extensive dorsal tegumental damage in the form of peeling of tubercles, spine disfigurement, vesicle formation and exposure of subtegumental tissue (×1500). **j** Higher magnification of tegumental changes (×4000). **k**, **l** LNCs deposited on the damaged subtegumental tissue and the loose disfigured spines (×30000). *Abbreviations*: F, female; M, male; OS, oral sucker; VS, ventral sucker; S, spine; T, tubercle; SP, sensory papillae; R, ridge; ST, subtegumental tissue; P, peeling of the tubercles

Results of the SEM examination are shown in Fig. 3. SEM images of normal *S. mansoni* male worms recovered from infected non-treated mice (NT) (Fig. 3a), showed round to oval oral and ventral suckers (Fig. 3b) with apically directed spines (Fig. 3c). The dorsolateral tegumental surface of the mid-body showed crab-like uniformly distributed tubercles that had sharp visible intact spines and sensory papillae (Fig. 3d). The tegument between the tubercles showed ridges (Fig. 3e). On the other hand, male worms recovered from *S. mansoni*-infected mice treated with the higher dose PZQ-MFS nanocombination (PZQ 250 mg-MFS 20 mg/kg, Subgroup Iab) showed deformity in the whole body (Fig. 3f) as well as in the oral and ventral suckers (Fig. 3g), with blunt, short and loose spines (Fig. 3h). There was extensive dorsal tegumental damage in the form of peeling of tubercles and spine disfigurement with appearance of subtegumental tissues (Fig. 3i, j). Marked deposition of nanostructures of the size of LNCs on the damaged tegument (Fig. 3k) and on the loose disfigured spines (Fig. 3l) was observed. The tegumental changes and sucker deformity observed in infected mice treated with either PZQ 250 mg/kg or MFS 20 mg/kg monotherapy (control Subgroups Ia and Ib, respectively) were almost similar. Comparable but milder morphological changes were also observed in worms recovered from infected mice treated with the lower dose PZQ-MFS LNCs combination PZQ 125 mg-MFS 10 mg/kg (Subgroup IIab) and the corresponding singly loaded LNCs (control Subgroups IIa and IIb).

#### Assessment of histopathological changes in the liver

**Fig. 4:**
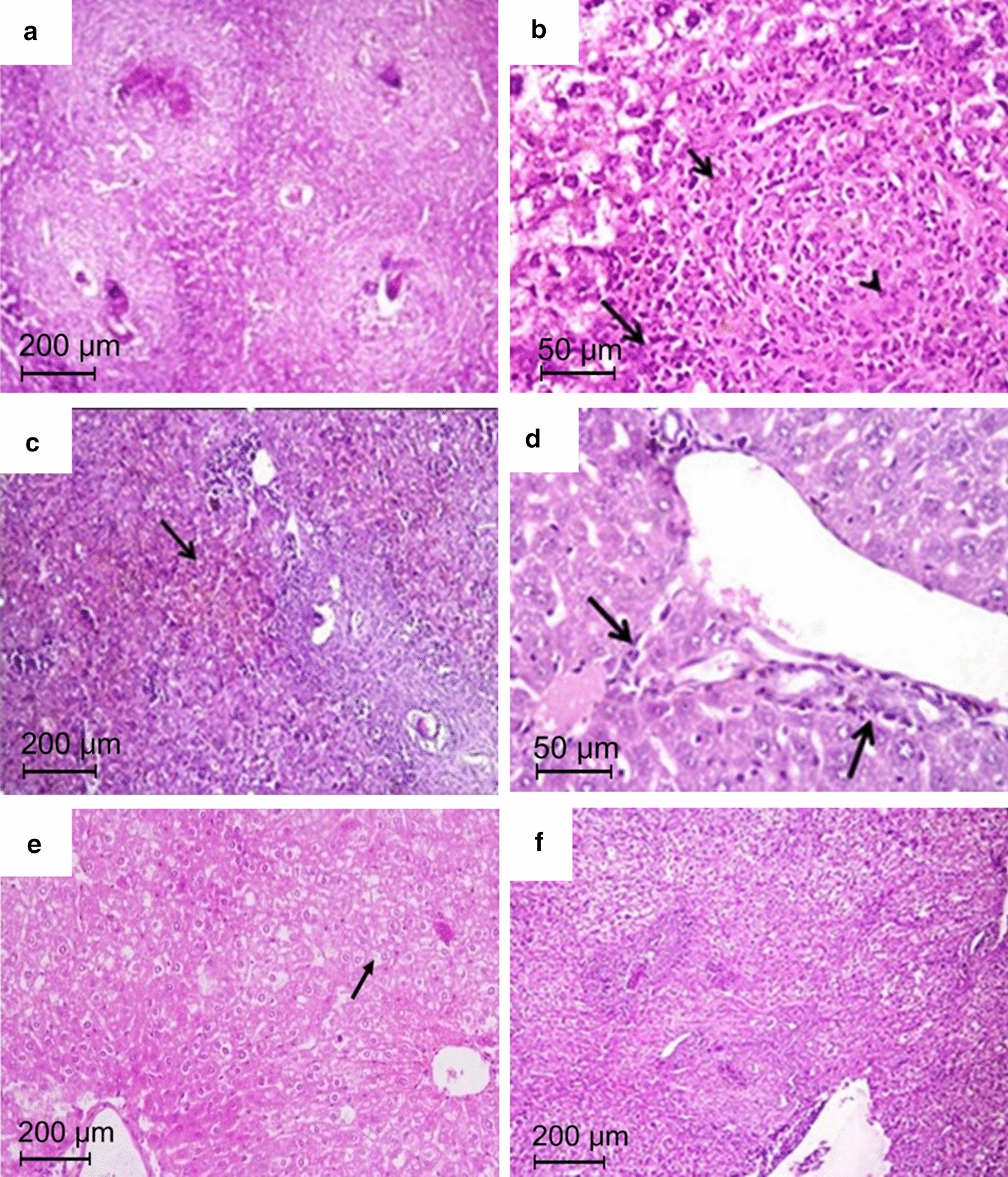
H&E-stained liver sections of *S. mansoni*-infected non-treated mice. **a** Preserved hepatic architecture and multiple numerous closely packed necrotic and exudative-productive granulomas (×100). **b** A granulomatous reaction formed of inflammatory cells mainly eosinophils (short arrows), neutrophils (long arrows) and histiocytes (arrowheads) (×400). **c** Brownish schistosomal pigment (arrow) (×100). **d** Kupper cell hyperplasia (arrows) (×400). **e** Fatty change of hepatocytes (arrow) (×100). **f** Liver section of *S. mansoni*-infected mice treated with the higher dose fixed combination (Subgroup Iab) showing small-sized healing granulomas (×100)

As shown in Fig. 4, the histopathological changes detected in H&E-stained liver sections of infected non-treated mice showed preserved hepatic architecture associated with granulomatous reaction located perivascularly and intraparenchymally (Fig. 4a). Most of the detected granulomas were active and formed mainly of eosinophils, lymphocytes and histocytes encircling laid *S. mansoni* eggs (Fig. 4b). There was deposition of schistosomal pigments (Fig. 4c), Kupffer cells hyperplasia (Fig. 4d), and fatty changes in hepatocytes (Fig. 4e).

On the other hand, pronounced amelioration of *S. mansoni*-associated hepatic pathology was observed in all developmental stages in infected mice treated with the higher nanocombination dose (subgroup Iab 1, 2 and 3), manifested as scanty small healing granulomas formed of minimal inflammatory cellular infiltration and surrounded by concentric collagenous fibrous tissue (Fig. 4f). Marked improvement of hepatic pathology was also observed in all infected mice treated with singly loaded control LNCs at the higher dosing level (Subgroups Ia 1, 2 and 3 and Ib 1, 2 and 3). On the other hand, only minimal improvement in infection-associated hepatic pathological changes was observed in all infected mice treated with the lower nanocombination dose (Subgroups IIab 1, 2 and 3) as well as their corresponding singly loaded control LNCs (Subgroups IIa 1, 2 and 3 and IIb 1, 2 and 3).

**Fig. 5:**
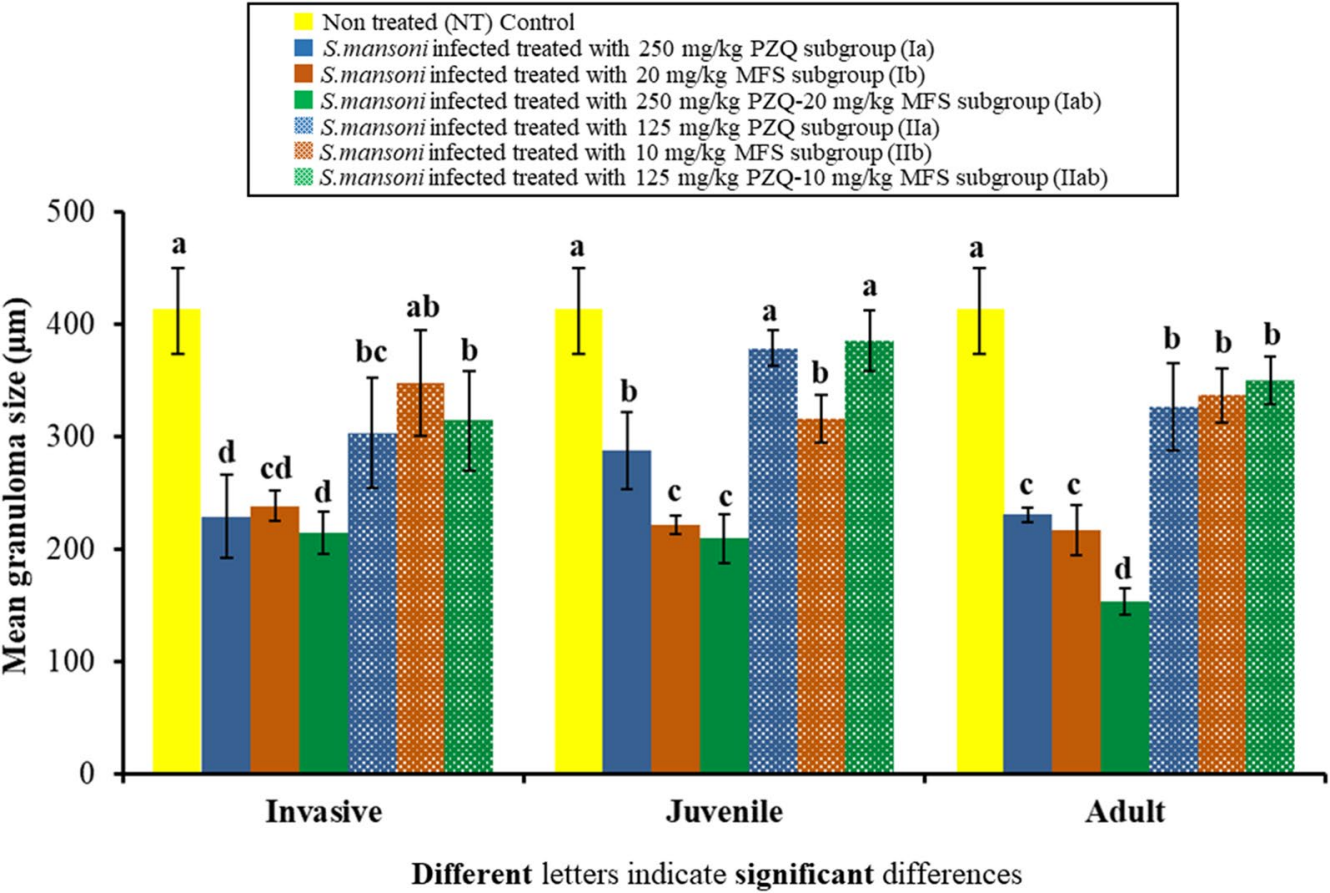
Effect of PZQ-MFS nanocombinations on hepatic granuloma size in comparison with non-treated and monotherapy controls against different developmental stages

Data for hepatic granuloma size in the study subgroups are shown in Fig. 5. There was a statistically significant difference between the different studied subgroups against invasive (*F*_(6, 35)_ = 21.877, *P* < 0.001), juvenile (*F*_(6, 35)_ = 59.525, *P* < 0.001) and adult stages (*F*_(6, 35)_ = 73.989, *P* < 0.001). Oral administration of the higher dose PZQ-MFS nanocombination (PZQ 250 mg-MFS 20 mg/kg, subgroups Iab 1, 2 and 3) to infected mice given against invasive, juvenile and adult worms resulted in a statistically significant reduction in the mean granuloma size (214.6 ± 18.47 µm, 209.6 ± 21.41 µm and 153.5 ± 12.0 µm, respectively) compared to a mean of 412.0 ± 37.83 µm for the infected non-treated control subgroup (*P* < 0.001). While the lower dose PZQ-MFS nanocombination (125 mg PZQ-10 mg MFS/kg, subgroups IIab 1, 2 and 3) given to infected mice against the three developmental stages induced a statistically significant reduction in the mean granuloma size (314.5 ± 44.01 µm, 385.4 ± 27.20 µm and 349.6 ± 21.26 µm, respectively) compared to the infected non-treated control subgroup (*P* = 0.001; *P* = 0.555; *P* = 0.003, respectively). On comparing the two different doses of PZQ-MFS LNCs (subgroup Iab and subgroup IIab) together, a statistically significant reduction in the mean granuloma size was observed in favour of the higher nanocombination dose given against the three different developmental stages (subgroups Iab 1, 2 and 3) (*P* = 0.001; *P* < 0.001; *P* < 0.001, respectively). There was also a statistically significant reduction in the mean granuloma size of mice treated with higher dose of PZQ-MFS LNCs (subgroup Iab) compared to PZQ-LNCs monotherapy given against juvenile and adult stages (subgroups Ia 2 and 3) (287.5 ± 34.46 µm and 230.6 ± 7.02 µm, respectively) (*P* < 0.001).

Statistical analysis of granulomas size of the two fixed-doses PZQ-MFS LNCs treated subgroups against the different developmental stages of *S. mansoni* worms compared to infected non-treated control, corresponding monotherapy controls as well as to each other are shown in Fig. 5.

## Discussion

We aimed at improving the antischistosomal therapeutic profile of PZQ in MDA programmes by enhancing multistage activity against the different developmental stages of the parasite, alleviating schistosomiasis-induced pathology and potentially reducing drug resistance while keeping dosage at a relatively low single oral dose. This cannot be achieved unless multiple strategies are exploited. In this study, an innovative approach based on the integration of the documented benefits of three strategies, namely drug repurposing, combination of multi-target drugs and nanotechnology, involved incorporation of PZQ and MFS in a nanocombination intended for single dose oral therapy of schistosomiasis mansoni.

As the key combination drug, PZQ exerts a direct antischistosomal effect on adult worms by underpinning the parasite Ca^2+^ homeostasis resulting in spastic paralysis and rapid vacuolization of the worm surface [44]. Recently, activation of a schistosome transient receptor potential channel by PZQ was shown to support the pharmacological profile of PZQ [45]. To overcome the known shortcomings of PZQ therapy, mainly ineffectiveness against immature worms and inability to ameliorate disease-associated pathology, MFS was included in the nanocombination. Selection of MFS relied on its current clinical use as the only approved oral drug for the treatment of visceral leishmaniasis [46] as well as earlier evidence of multistage antischistosomal activity of oral MFS in mice [13]. Although the antischistosomal mechanism of action of MFS is not well established yet, antiparasitic and anticancer effects of MFS appear to be related to its high affinity for plasma membrane [47, 48]. As a membrane active zwitterionic alkylphospholipid, MFS causes dramatic increases in membrane dynamics by interacting with the protein component and inhibiting phospholipid turnover and lipid-dependent cell signaling pathways, leading to apoptosis. Of importance to schistosomiasis, MFS impedes the biosynthesis of sphingomyelin, a schistosome membrane phospholipid which hides the surface membrane proteins from the host immune system [49]. Evident damage to the *Schistosoma* tegument with exposure of their surface antigens was reported following treatment with MFS [13, 16, 50].

Selection of LNCs as nanocarrier for the oral administration of the PZQ-MFS combination was based primarily on its recognized merits as lipid-based nanocarrier for oral drug delivery with modifiable composition. For example, LNCs were demonstrated to enhance the activity of MFS against *S. mansoni* compared to the free drug (67.51 *vs* 8.13% reduction in worm burden by MFS LNCs and free MFS respectively) with further enhancement (88.46% reduction in worm burden) upon modification of LNCs with oleic acid as membrane permeabilizer [16]. Owing to their amphiphilic nature, MFS molecules are possibly intercalated within the tensioactive shell of LNCs, enhancing their structural integrity and allowing for potential translocation across the intestinal membrane. A hypothesized structure of MFS-LNCs has been reported earlier [16]. LNCs modified with oleic acid and MFS as formulation additives also enhanced the activity of PZQ against experimental schistosomiasis mansoni [24]. In both MFS and PZQ systems, LNCs allowed marked sustained release of the active drugs for at least 24 h.

Analysis of data obtained in the present study indicated that the activity of PZQ LNCs (250 mg/kg) against schistosomiasis mansoni was in the order of adult > invasive > juvenile while that of MFS LNCs (20 mg/kg) was in the order of juvenile > adult > invasive corroborating literature data for both drugs in the free form [13, 51, 52] and nanoencapsulated form [16, 17, 24]. The nanocombination of MFS with PZQ in the same doses (PZQ 250 mg/kg-MFS 20 mg/kg) resulted in high activity against prepatent and patent infection implying multistage activity, a main objective of the present study.

Reduction in mean adult worm burden (Fig. 2), the amelioration of hepatic pathology (Fig. 4) in addition to liver granuloma size (Fig. 5) were obvious in the higher dose nanocombination-treated group (Group Iab) compared to the infected non-treated control and the respective singly loaded PZQ monotherapy treated subgroups and in particular when given against the juvenile stage (Subgroup Ia 2). This denoted greater amelioration of schistosomiasis-induced pathology which results from immune-mediated granulomatous responses against *Schistosoma* eggs trapped in tissues. These are likely to cause serious local and systemic pathological effects associated with granuloma formation and fibrosis [53]. The significant reduction in hepatic granuloma size could be attributed to the distribution of PZQ [13, 54] and MFS [55, 56] to the liver of mice. Hepatic granulomas shrink progressively after PZQ treatment because of schistosome killing and reduction in the number of eggs trapped in the liver, reversing fibrogenesis [57].

In addition, adult male schistosomes recovered from *S. mansoni*-infected mice in the higher dose nanocombination subgroup (Iab) showed deformed suckers as well as extensive tegumental damage in the form of peeling of tubercles, disfigurement of the spines and appearance of subtegumental tissue (Fig. 3) supporting earlier observations for singly loaded PZQ LNCs [24] and MFS LNCs [16]. Suckers promote the attachment of schistosomes to blood vessels for the feeding process [1]. The tegument of *Schistosoma* also plays an important role in the uptake of nutrients and ions, excretion of metabolic end products as well as protection against the host immunological attack [58].

The obvious change observed in the therapeutic profile of nanoencapsulated PZQ induced by the addition of MFS in the higher nanocombination dose pointed to the benefit of combining drugs having different modes of action in appropriate dosing. This substantiated literature data for antischistosomal combinational therapies based exclusively on free drugs to date. Examples of PZQ combinations with enhanced efficacy in terms of reduced worm burden, or protection against pathological sequelae or multistage activity include PZQ-artesunate [59], PZQ-mefloquine [60, 61] and PZQ-edelfosine [62]. However, the fear of development of *Plasmodium* resistance to antimalarials and the relatively long treatment duration required for other combinations may hinder their applicability. This highlights the benefit of the current single oral dose nanoformulation.

Another great advantage of antischistosomal LNC formulations is their tegumental targeting ability observed earlier for MFS LNCs modified with oleic acid [16] and PZQ LNCs modified with oleic acid and a low concentration of MFS as formulation additives [24]. Such observations have been substantiated in the present study by high magnification SEM visualization of nano-objects deposited on the damaged tegument and the loose disfigured spines of adult *S. mansoni* worms (Fig. 3k, l). The size of these nano-objects was close to that of LNCs (57.61 ± 0.15 nm). Noteworthy, no such nanostructures were observed for free unencapsulated MFS [13, 15] nor PZQ treatments [24]. Findings regarding these nano-objects corroborate published data documenting possible intestinal translocation of LNCs across the intestinal wall [29, 63]. Combined data emphasize the role of oral nanocarriers in mediating cellular drug delivery, providing new avenues for modified therapies [64].

A further objective of the study was to keep the doses of PZQ and MFS at the lowest level for effective therapy. Although the amounts of PZQ and MFS entrapped in the higher dose PZQ-MFS nanocombination were already reduced relative to the free drug forms [16, 43], an attempt was made to assess the effect of halving the dose of both drugs on the combined antischistosomal activity of a second lower fixed-dose nanocombination using the same procedures (Figs. 2, 5; Group II). Results indicated a statistically significant reduction in the antischistosomal activity of the lower nanocombination dose and its singly loaded control counterparts compared with the corresponding higher dose formulations.

Based on the results obtained, the antischistosomal activity of PZQ as the gold standard of antischistosomal therapy to date could be significantly enhanced by utilizing a multiple approach based on drug combination, drug repurposing and nanotechnology. Interaction of the nanocombination components resulted in the great promises offered by the higher dose PZQ-MFS nanocombination developed in the study. These include high efficacy against different developmental stages of experimental schistosomiasis mansoni infection at the reduced doses of 250 mg/kg of PZQ and 20 mg/kg of MFS. Efficacy enhancement was bestowed by the repurposing candidate, MFS, which also contributes to the structural stability and membrane activity of LNCs, probably facilitating tegumental targeting. Moreover, a statistically significant reduction in the size of hepatic granulomas, implied amelioration of a serious disease-associated liver pathology. Finally, a rationally selected nanocarrier (oleic-acid modified LNCs) showing controlled drug delivery and great benefits in oral administration contributed to efficacy enhancement by facilitating the intestinal translocation of the nanocombination and tegumental targeting.

## Conclusions

The fixed-dose of 250 mg PZQ-20 mg MFS/kg nanocombination developed in the present study using LNCs offers advantages as a potential oral nanomedicine for single dose antischistosomal therapy with multistage activity and ability to ameliorate schistosomiasis-associated hepatic pathology. The study findings provide the first evidence on the benefits of integrating drug repurposing, combination therapy and nanotechnology as a novel multiple approach to improve the therapeutic profile of PZQ. Findings also verified the merits of LNCs as oral nanovectors and a carrier system for antischistosomal drug delivery, expanding their biomedical applications. From a clinical standpoint, utilization of the fixed-dose PZQ-MFS LNCs in MDA campaigns may offer great therapeutic potentials including a simpler administration process and radical cure allowed by multistage activity. Further studies are needed to document the safety of the developed nanocombination and explore its prophylactic activity, potential to hinder the onset of resistance to the differently acting drug components as well as cost effectiveness.

## Data Availability

The data supporting the conclusions of this article are provided within the article.

## Abbreviations

EE%: entrapment efficiency
GI: gastrointestinal
H&E: hematoxylin and eosin
LNCs: lipid nanocapsules
MDA: mass drug administration
MFS: miltefosine
PCS: photon correlation spectroscopy
PdI: polydispersity index
p.i.: post-infection
PZQ: praziquantel
SEM: scanning electron microscopy
TEM: transmission electron microscopy
ZP: zeta potential
